# Editorial: Expert opinions: save the microbes to save the planet

**DOI:** 10.3389/fmicb.2025.1736820

**Published:** 2025-12-09

**Authors:** Rino Rappuoli, Anna Maria Puglia, Mariagrazia Pizza

**Affiliations:** 1 Fondazione Biotecnopolo di Siena, Siena, Italy; 2 Dipartimento Innovazione, Consorzio Italbiotec, Milan, Italy; 3 Centre for Bacterial Resistance Biology, Imperial College London, London, United Kingdom

**Keywords:** microbes, climate change, IUMS Congress, sustainable solutions, planet health

The industrial revolution provided huge benefits to humankind and changed the course of history. However, the growing demand of energy to sustain machines, that is mostly based on fossil fuels, started a slow but irreversible increase of the temperature of our planet. In 2024 the temperature reached for the first time the record high of 1.5 °C above that registered before the industrial era. This temperature increase is mostly due to the accumulation in the atmosphere of greenhouse gases such as carbon dioxide, methane and nitrous oxide, which do not allow the heat of the sun to escape from the atmosphere. The increase of the temperature of the planet is already causing dramatic effects: tropical diseases are moving to Southern Europe and North America; extreme weather events are devastating the continents with increased frequency; millions of tons of plastic are polluting soil and oceans and releasing microplastics that contaminate the cells of most living organisms; production of food for eight billion people is done utilizing chemical fertilizers which pollute the soil, rivers, lakes, and oceans; and, deforestation and urbanization are creating new diseases and new pandemics.

We are getting very close to the point of no return, and we have the urgency of adopting sustainable solutions, because the human population cannot be healthy unless the planet is also healthy. Microbes created life on Earth. The first bacteria appeared approximately 3.8 billion years ago, they generated the oxygen that is present in our atmosphere, they generated all living organism, including plants and animals.

One trillion species of microbes sustain the life of our planet; they live everywhere, including most extreme environments. They evolved a life on the planet based on sustainable solutions and have the power to provide solutions for most of the causes of climate change. Microbes can digest greenhouse gases and use them to make fuels and chemical compounds; they can digest plastics and generate biodegradable plastics; they can produce natural fertilizers by fixing nitrogen from the atmosphere; and they can provide food to the planet without the use of chemical fertilizes. Microbes can use biological wastes to generate energy, heat, fuels, and chemical compounds. All compounds that today are made by burning fossil fuels, in theory can be produced without pollution by microbes.

The main reason why we have not paid attention to the power of the microbes is because we invested so much on burning fossil fuels, and we created industrial plants based on fossil fuels, so that today it is very convenient to continue with them. However, we have reached a point where, if we care about the future of the planet and about the future generations, we have no choice. We need to find sustainable and responsible solutions for our planet and these solutions cannot be found without the help of microbes. For this reason, the International Union of Microbiological Societies (IUMS) decided to make climate change a major focus of modern microbiology, by launching a call for action, inviting all the microbiologists of the planet to think how they can contribute to the health of the planet by developing microbe-based solutions to address climate change. The call for action was published in 2023 (Rappuoli et al., 2023) and was followed up by several other publications (Crowther et al., 2024; Rappuoli et al., 2025; Borgianni et al., 2025; Lennon et al., 2025). The topic was also discussed in a symposium and a dedicated panel at the IUMS meeting held in Florence in October 2024. On that occasion, it was decided to provide the opportunity to all microbiologists worldwide to contribute to the topic by submitting papers to this issue of *Frontiers in Microbiology*. The papers in this volume, while they do not provide yet final solutions, report the beginning of a new era where microbiologists will be major players in addressing the health of the planet by discovering and developing microbe-based sustainable solutions.
